# *Data Domotopia*: introduction to the quantitative survey

**DOI:** 10.1007/s11116-023-10388-y

**Published:** 2023-04-26

**Authors:** Marc-Edouard Schultheiss, Fiona del Puppo, Garance Clément, Guillaume Drevon, Vincent Kaufmann, Luca Pattaroni

**Affiliations:** 1 Laboratory of Urban Sociology (LASUR), Ecole Polytechnique Fédérale de Lausanne (EPFL), Station 16, Lausanne, CH-1015 Switzerland; 2 Morgan Center, The University of Manchester, Oxford road, Manchester, M13 9PL UK

**Keywords:** Immobility, Stillness, Resilience, Action space, Time studies, Home-making, Support network

## Abstract

This paper describes the *Data Domotopia* a 2300 + respondent self-administered web-based survey. It includes 100 + multi-purpose items about home-making and stillness in a moving world. We suppose that home-making can reveal coping strategies and resilience practices to make everyday life work – as home is a central location in people’s activity-travel patterns. To describe this phenomenon, the concept of *Domotopia* is introduced, defining how people arrange, use, and experience their homes to cope with the pathologies of accelerated and liquid modernity (Bauman 2005). While the Data Domotopia is based on a mixed-method combining qualitative and quantitative material, this paper focuses mainly on the description of the questionnaire – which is organized into three interrelated layers: the dwelling, the dwellers, and the neighborhood. Each of these layers unfolds in functional, social, emotional and sensory components. The survey covers most of the contemporary issues related to home-making. This includes the domestic space and gender issues; the socio-spatial resources (mobility, action space, core, and wider social network); lifestyles, ideals, and residential aspiration; time pressures, time use, organization and stress; equipment, rules and arrangements; interpersonal relations, cohabitation and negotiation, dominance and power. Intakes on the *Data Domotopia* is given by two concrete cases about the time-space coverage of the habitual action space, and about inter-personal task allocation. These examples show the potential of the data to study domocentric stillness and resilience to urban pathologies. The data – aggregated to the infra-communal level – is available for research purposes.

## Introduction

With the prevalence of technological optimism (Morozov 2014) in the development of sustainable cities; and in a context of high mobility as a commonplace and widespread social phenomenon (Kaufmann and Viry 2015), the modern ways of living are confined to man-made, anthropocentric and increasingly uncontrollable system of systems (Latour 2021; Morin 2021). A recent line of research highlights the relevance of immobility as a source of resilience to cope with this system dependence (Ferreira et al. 2017). Different concepts of resilience exist (e.g., Dovers and Handmer 1992; Walker et al. 2004). Here, the term resilience is understood as a capacity of resistance to uncertainties, adaptability, and transformability to disruptions or alterations. Most of the literature about immobility relies on the rationale that mobility dependence cannot be ignored anymore from future scenarios (Larsen et al. 2006; Sheller and Urry 2006). Thus, transport policies and research must consider immobility seriously (as defended in the first chapter of “Stillness unbound” by Fuller and Bissel, 2013). In travel diaries or time-use surveys, a high number of people usually do not declare any trips or activities, yielding shares up to 24% in recent European time-use surveys (Madre et al. 2007). The trustworthiness of these results can be discussed as a significant part – varying across surveys – may originate in the survey protocol itself that led respondents not to declare their travels as a strategy to lower the response burden (Huber et al., 2008). Further analysis suggests that only a range of 8–12% of people are immobile in a working day period, while on the other hand, a growing portion of the population has become captive to high mobility patterns in everyday life. The diversification of ways of living makes society more dependent on urban systems (Urry 2014). This can easily introduce friction in people’s lives (Cresswell 2010) – as mobility involves a fragile entanglement of physical movement, collective representations, and social practices (Kaufmann et al. 2004).

In this sense, immobility can foster a form of resilience for the transport system itself – as described by Ferreira et al. (2017, p. 17) – but also, and more broadly, a form of resilience in the orchestration of the different spheres of life: Immobility as a source of resilience to cope with the pathologies of a liquid and fast-changing society (Bauman 2005).

From this perspective, it has become a privilege to be able to pursue slowness, stability, or stillness. Permanent movement and incessant change lead to undesirable time pressures and stress. From a territorial perspective, immobility translates into new forms of localism and proximities, such as the compact city, the slow city, the vital city, the 15-minute city, and so on. However, despite these promising developments, immobility remains largely overlooked in the literature. Firstly, because of data availability. Immobility is generally considered as the absence of mobility; immobility is rarely the main focus in data collection campaigns (Lucas and Madre 2018). It creates measurement issues in travel surveys or time-use surveys (Hubert et al. 2008; Madre et al. 2007). The second reason why immobility happens to be overlooked in the literature is the common assertion that immobility has a negative connotation, that it is something endured, often correlated with financial poverty or illness, and synonymous with idleness and stagnancy. These conceptions disregard immobility as a deliberate choice. Individuals are in a perpetual cycle of mobility and immobility (Cresswell 2010). Immobility is not a marginal phenomenon (Madre et al. 2007). Moments of immobility offer a breathing space in activity-travel chains, and “a day without travel does not mean a day cut off from the world” (Motte-Baumvol et al. 2022). This is particularly relevant in our modernity, in which working from home is more and more common, and access to services and culture from home is more and more facilitated. Going further, “immobility may just as easily reflect a situation in which individuals are free to use their time as they wish as it may reflect an inability” (ibid., p. 4). Leveraging two waves of the United Kingdom National Travel Survey, recent empirical research showed that the level of time and space constraints is a major vector of immobility (ibid.). Thus, relaxing the time and space constraints offers more flexibility that people use being immobile. Yet, we have to slacken the idea that being immobile reflects a situation in which individuals are free to use their time as they wish. Such *a priori* would render invisible all the tasks performed in the domestic sphere, and all the stress and pressures related to home-making, or interpersonal organization with relevant others.

In this paper, we describe the dataset *Data Domotopia*, a 2300 + respondent, a self-administered web-based survey consisting of 100 + multi-purpose items about “home-making in a moving world”. We assume that home is a central location of immobility in people’s activity-travel schedules and that home-making can reveal coping strategies and resilience practices in terms of resistance, adaptability, or transformability to make everyday life work, in particular, to cope with exhaustion, burn-out, stress, anxiety, the pathologies identified as related with accelerated life rhythms and liquid modernity (Antonioli et al. 2021).

A home is a place where household members cohabit and synchronize. Where time is slower, or sometimes faster. A home reflects dwellers’ aspirations, and ideals in terms of ways of living or organizational strategies. A home is a life world, a place of emotions, a place where letting go is allowed. A home is a sanctuary or a hyper-place where people, information, and communications converge. Besides, in the context of social acceleration and liquid modernity, the home must now accommodate new functions rooted in different spheres of life (Lussault 2017). The use of private spaces, resting time, or leisure time tends to diversify. A home is a place to work, play, consume, to socialize beyond close family and friends. A home is also a place where domination and power issues play out.

*What does “home” mean and how is the home used in an ever-fast-paced world*.

*How are the inequalities in domestic life transformed by time pressures?*

*What role do the home play in daily life and activity-travel chain?*

Given all the intricacies of the pathologies and the resilience previously introduced, this data paper describes a questionnaire that was designed to understand how people make use of, experience, and relate to home-making given the pace of their daily activities. Consider that three main standpoints are considered. The *dwelling*, as the architectural object (with various forms and morphologies but also economical weight and characteristics), where the *dwellers* develop a sense of place in its sensitivity and intimacy (Rodaway 1994); and which is part of a broader *context* including the spatial, social, and the temporal dimensions of the extended habitat. The *Data Domotopia* focuses on the residents of the Canton of Geneva, Switzerland, and was administered in 2021. A translated version of the questionnaire is available online (see Pattaroni et al. 2022).

The remaining text is structured as follows. Section 2 motivates the questionnaire by its theoretical background and depicts other quantitative surveys addressing related topics. To the best of our knowledge, no prior examples of similar surveys exist. Section 3 develops the research methods, introducing the analytical grid used to build the questionnaire. Also, insights on a series of qualitative interviews conducted in parallel with the questionnaire are provided. Section 4 outlines the main themes of the questionnaire. Section 5 provides insights into the available data, developing two concrete use cases on time-space coverage of the habitual action space on the one hand, and the inter-personal task allocation problem on the other hand. Lastly, Sect. 6 briefly concludes the data paper and calls for further contributions – as *Data Domotopia* is made available upon request and will be archived by the end of 2023.

## Background

The dataset described in this paper – the *Data Domotopia* – contributes to the emerging interest in urban rhythms, time use studies, and rhythms of social life (e.g., from a transport engineering perspective with Schönfelder and Axhausen 2010; or a social science perspective with Drevon et al. 2019). As developed in the literature of Zygmunt Bauman, Paul Virilio, Michel Lussault or Hartmut Rosa (Bauman, 2005; James, 2007; Lussault, 2017; Rosa, 2013), speed, acceleration and liquidity appear in the shortening, fast-changing and densification of experiences. In this context, we assume that the home is one of the last space-time locations where immobility is possible and allowed. Home – or housing, or home-making – constitutes a promising prism to study immobility, together with its forms of resilience to cope with modern pathologies such as the hybridization of activities, and the complexification of work-life balance.

While the temporal dimension is of long-lasting interest for the analysis of modern societies (e.g., Nowotny, 1996; Pentland et al., 2002; Wajcman 2014), it seems important not to disregard the spatial dimension of social rhythms, and in particular the resources of stillness and immobility in a mobile world (Fuller and Bissell 2013). The concept of *Domotopia* considers the intimate constitution of home-making together with its increasing connection to satellite spheres of life. Domotopia was forged as an analogy to what Michel Foucault (2009) defined as *heterotopia*: an “other space” with both isolation and openness characteristics, reaffirming the importance of space in social dynamics (Soja 1989). In the continuity of this approach, the philosophy of the *Data Domotopia* is to focus on the socio-spatio-temporal state of modern society emerging and made visible in home-making through the triptych “*dwellers*, *dwelling*, and *context*.”

### Dwelling

The attribution of specific uses to different rooms in a dwelling establishes a direct equivalence between time and space (Amphoux et al. 1989). From this perspective, the dwelling layout can be read as the spatialization of located temporalities. In the 19th century, the bourgeoisie considered housing as a privileged space for intimate and family life. This ideal has encouraged an architectural specialization of the dwelling spaces. It resulted in residential / apartment plans with strong separation between spaces for social demonstrations on the one hand, and spaces dedicated to privacy and withdrawal on the other hand. The moral order based on the nuclear family and supported by this bourgeois domestic architectural ideal was later spread to more popular homes and households (Eleb 1990). Conversely, the contemporary habitat has blurred these separations, to the point where even the most intimate spaces – such as the bed – are connected to the outside world (Tapie 2014). The limits of private spaces are not only blurred by means of screens and media but also by the return of cleaning, care, and domesticity professionals – who had progressively disappeared from most home interiors since the beginning of the 20th century –, the possibility to consume from home, and the adoption of smart devices (de Maat 2015). We assume that these new home-related liquidities go along with new housing appropriations and equipment, new ways of living, inhabiting, and cohabiting. The transformations of domestic life must therefore be questioned, using both spatial and social factors to understand how emancipation and resilience now occur in the dwelling.

### Dwellers

Beyond its functionality and spatiality, the dwelling is a complex social space. It holds a statutory role (e.g., as simple as a postal address, see Fijalkow 2022), constitutes and shapes the class, gender, and lifestyles of the dwellers (e.g., bourgeoisie in Pinçon-Charlot and Pinçon 2018; workers in Schwartz 2012; or more about gender in Gilbert 2016). The social process of home-making also implies an “active” dimension, as it implies the self-construction of a lifeworld and appropriations. The latter is key in the pursuit of resilience in the capacity of adaptability and transformability (Serfaty-Garzon 2003). Inhabiting also implies cohabitation, which raises various forms of political issues, inequalities, and domination (Barthes 2012). The dwelling is also a complex social space because there is a discrepancy between the socialization of the dwellers, their resources, and the pace of societal evolutions and changes in the temporalities of contemporary society. More women get to work full-time, there is a noticeable increase in single-parent households, and the youngest household members spend more time in higher education which postpones emancipation. As a consequence, the roles within the domestic sphere are changing, maybe faster than the roles in the professional sphere. Or the other way around. This phenomenon creates important inequalities regarding the ability to enjoy free time, often limited for those who cover most of the domestic tasks of caring, cleaning, or maintaining (Devetter and Rousseau 2011). In particular, the assignment of those tasks heavily relies on gendered assigned roles (Molinier 2012). The destandardization of the workday complexifies the synchronization of individual, familial and social activities within the home (Lesnard 2015), and brings in the home the time pressures originating from the professional sphere (Aubert 2006).

### Neighborhood, context

Housing is part of a broader action space, characterized by temporal and locational habits. The organization and time management directly relies on the geographical size of the action space, the morphological opportunities (e.g., quality of the transport network), and the proximity to urban functions (e.g., facilities, services, transit network). Housing is also part of a social fabric, a sense of place (Felder 2021), a system of “milieux”, and a support network such as friends, family, or neighbors. More recently, home-making has also become part of a digital fabric, equipped with connected devices and remote services. Transportation and communication systems are transforming the ways of living by altering the perception of proximity and residential opportunities (Kaufmann 2014). For example, new residential practices emerge because of the new possibilities of doing things remotely (e.g. multi-tasking while commuting, or bi-residentiality). Thus, the action space and the social support network are a third key resource of resilience in home-making. However, it must be stressed that the urban, social, and digital fabrics are also sources of injunctions to mobility, social pressures and inequalities, or forms of segregation (e.g., Bonvalet and Dureau 2000).

### Related surveys

The next section reviews selected data sources where one can get information about the different themes and topics identified above. For the context, we searched for datasets describing the action space, the organization and time management, the social and support network, and the equipment and functional resources. For the dwelling, we searched for information about the housing architectural features, and housing conditions, uses, appropriations and aspirations, residential mobility, and housing career. For the dwellers, we searched for household structures, repartition of domestic and care tasks within those households, coupling constraints, interpersonal plays of power, and time pressure pathologies. This review is synthesized in Table 1, which cross-references the aforementioned topics with what is available in open data, time-use surveys, travel diary surveys, household panels and family surveys, and living conditions surveys. A few methodological elements are also specified as to whether the data source presents longitudinal or cross-sectional data, single- or multi-person data, and whether the respondents are sourced from a panel.

**Open data** include freely accessible information about the urban forms (e.g., OpenStreetMap, 2022) as well as official statistics like the building and dwelling statistics (e.g., FSO, 2019). In the past few years, a large effort has been made to increase the quality of open data, push the threshold of what can be open, and maintain the data up to date (see for example in Switzerland the actions of the Open data association, 2022; and the data platform of SBB, 2022). Open data can complement the survey data and open up vast analytical possibilities. In particular, open geospatial data describing urban morphologies are now readily available with numerous attributes – see the web platform of SITG (2022b) for a comprehensive open data collection and the doctoral thesis by Schirmer (2015) for intakes about the classification of the urban morphologies.

**Time use surveys** are generally conducted to “quantify how much time people spend on various activities, including paid work, household chores, and family care, personal care, voluntary work, social life, travel and leisure” (eurostat, 2020). Time-use surveys often ask for declared time pressure pathologies, like the feeling of stress, or the feeling of lack of time. If one example had to be cited, the Harmonised European Time Use Survey (HETUS) is based on a household questionnaire, an individual questionnaire, and a time-use diary. It is conducted only once a decade (in 2000, 2010, and 2020), but across 18 European countries. Additionally, novel survey methods and tools for obtaining rich longitudinal accounts of individuals’ travel and time use are emerging. For example, the TimeUse + diary mobile app (see Winkler et al. 2022) collects GPS tracks passively over several weeks and has users actively enrich tracked events with activity information. This longitudinal approach is useful to capture the variability and habits in travel and time use behavior, as well as more accurately capture activity space sizes.

**Table 1:** Review of topics covered by overlapping surveys (non-exhaustive)

		Open data	Time use survey	Travel diary survey	Household panels / family survey	Living conditions / dwelling statistics	Data domotopia
*CONTEXT*	Action space	x	-	x			x
	Organization and time management		x	x			x
	Social / support network			-			x
	Equipment and functional resources	-		x	-	x	x
*DWELLING*	Housing forms, layout and conditions				-	x	x
	Housing uses, appropriation, aspiration				-	x	x
	Residential mobility / housing career				x	x	x
	Domestic tasks and care		x		x		x
*DWELLER(S)*	Respondent profile	x	x	x	x	x	x
	Household structure	x	x	-		x	x
	Interpersonal constraints and powers		-	-	x		x
	Time pressure pathologies		x			-	x
METHOD	Longitudinal / retrospective data		-		x	x	
	Cross-sectional		x	x		-	x
	Multi-person		-		x	x	-
	Panel				x	-	

x: *included* -: *sometimes included with exceptions, or can be derived*

**Travel diary surveys** are designed to collect information on how, why, when, and where people travel as well as factors affecting travel. They provide mostly self-reported information about the available mobility tools, trip purpose, mode of transport, and time of travel. Information is usually collected over a single day of observation, for a single person in the household, and is mainly leveraged for transport planning. Rarely, do some surveys provide multi-day or multi-person information (e.g., Axhausen et al. 2002). Activity-travel data generally allows to derive details about the action space, scheduling, and organization, and often household structure can be derived. However, it remains complicated to explore the coupling constraints with relevant others in-depth, and moments of immobility are difficult to interpret (Madre et al. 2007). Mainly, travel diary surveys overlap with the Data Domotopia in the description of the action space, system of milieux, scheduling and activity organization, and sometimes information about the social support network. Among the most used datasets in Europe, the United Kingdom National Travel Survey (i.e. multi-person and multi-day) that is conducted each year (GOV.UK, 2022) as well as the German mobility panel (MOP), are based on 7-day travel diaries household surveys, with an extra longitudinal dimension provided by 3 consecutive years of answers for each participating household in the MOP case (mobilitaetspanel.ifv.kit.edu, 2022).

**Surveys on living conditions** are very similar to the *Data Domotopia* in the sense that they collect a wide variety of topics providing both an objective and subjective look at the multidimensional aspects of living conditions. They are designed to collect information about the social and economic conditions of life and allow a better understanding of what influences them. It includes in particular information about social exclusion, housing (type of dwelling, housing conditions, cost), values and satisfaction with living conditions, sense of security, social relations, and childcare. Surveys on living conditions are part of a European effort since very similar surveys are conducted in several European countries (e.g., eurostat, 2021; FSO, 2021).

Lastly, **Household panel and Family surveys** cover a broad range of fields and a variety of topics including information on sociodemographic, economic, psychosocial, life courses and perceptions. Household panels can present as one-time surveys, such as the ones recently conducted in Germany (Microcensus, 2021) and France (INSEE, 2009). These protocols can provide extensive information about children, powers and negotiations of couples, domestic tasks, and care. These surveys overlap the *Data Domotopia* mainly in the intra-household structure, conditions and negotiations. Several other household panel surveys are longitudinal and multi-person: they can make visible the mutual influence of household members’ attitudes and behavior over time. This type of longitudinal household panel have been conducted nationwide in Germany (SOEP, 37 times between 1984 and 2020) and Switzerland (every other couple of years for the past three decades (e.g., EUI, 2022; FORS, 2022; ISER, 2022).

To conclude, data domotopia brings a unique contribution by standing in the middle of these existing surveys (time-use, travel diary, living condition, and household surveys). This mixture provides an original look at the multidimensional aspects of home-making and domocentric immobility while preserving overlaps with existing data to ease the dissemination and interpretation of the data. This includes travel, action space and support network, conditions of the dwelling, family and domestic configurations, the pace of life, and time pressure pathologies. In addition to the new links created between the related surveys, the overlap analysis (Table 1) reveals that the Data Domotopia brings not-so-common contributions regarding time pressure pathologies, social network (friends, family, or paid resources), and interpersonal constraints, powers, and negotiations.

## Method

This section provides details on the methodology of the study. It summarizes the logic of the questionnaire – as presented in more detail in Sect. 2 – and elaborates on the details of data acquisition and collection. It also provides insights into the mixed-method approach. Indeed, *Data Domotopia* is part of a larger project based on a quantitative-qualitative mixed-method analysis to explore home-making in its plurality. The quantitative part remains the main focus of this paper. However, the qualitative approach complements the questionnaire and it appeared relevant to highlight the importance of such mixed approach.

### Questionnaire rationales

**Fig. 1:**
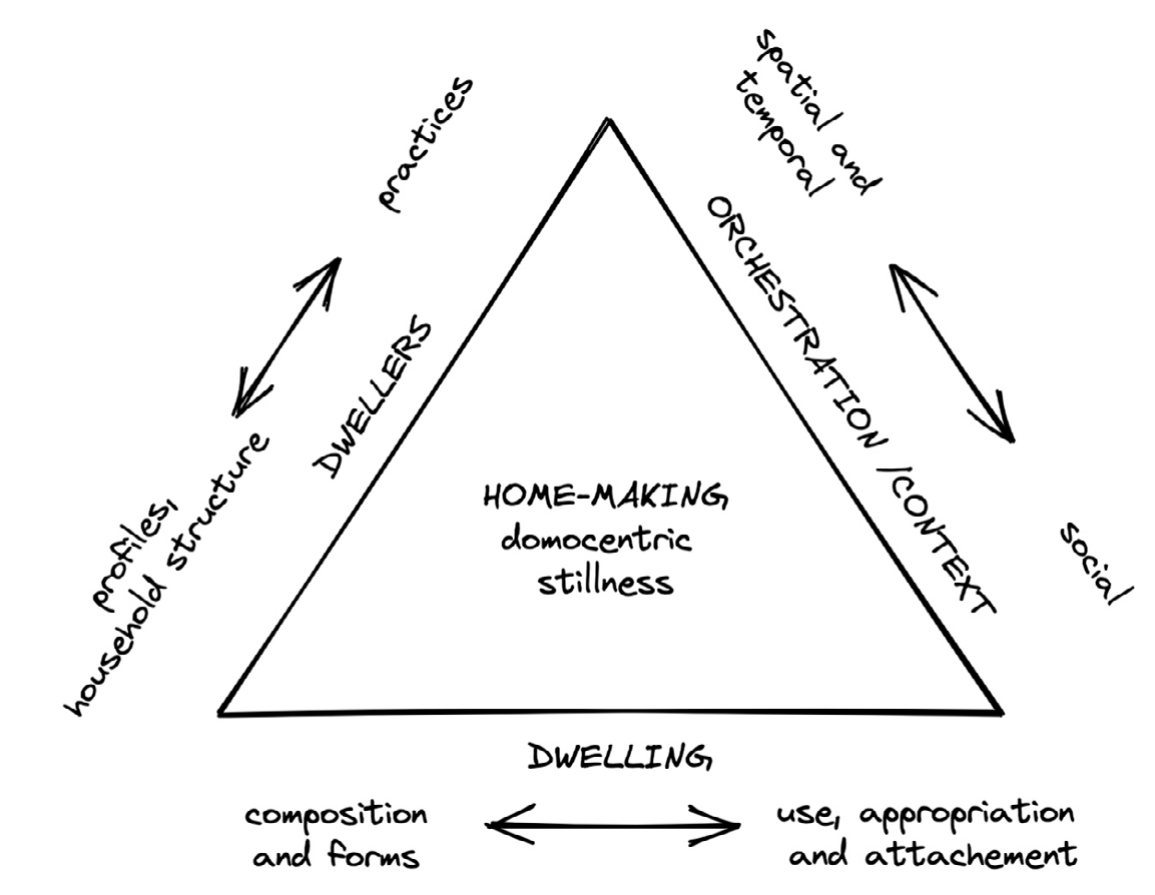
Methodology of the Data Domotopia

Based on a synthesis of the theoretical background, a threefold methodological device is proposed in Fig. 1 to describe the *Data Domotopia*. First, home-making is seen through the dwelling, ranging from its morphologies (e.g. composition, layout, forms, equipment) to its various forms of appropriations (e.g. uses, spatial organization, temporal mixing, emotional attachments). The objective is to verify to what extent the dwelling can be a place of sanctuary i.e. where people seek calm or isolation; or a hyper-place i.e. where people over-communicate and over-socialize. Second, home-making is seen through the dwellers. The dwellers are traditionally characterized by demographics and socio-economics, or by a familial configuration. Here, it is to study the interpersonal relations within the household and to reveal dominances and powers, gender effects, inequalities, or other forms of constraints and pressures happening in the everyday life of households. Third, the dwellers and the dwelling are put in the perspective of their social and geographical contexts – the familiar public spaces, the temporal and locational habits, the social life of the neighborhood, the support network, the activity space, and other spheres of life.

The way respondents organize these elements in their everyday life is also a recurrent theme of the questionnaire. The research device displayed in Fig. 1 provides an analytical grid for the *Data Domotopia*, which always returns to the central issues of home-making and the resilience revealed by domocentric stillness.

### Mixed-methods

From a broader perspective, the method underpinning the Data Domotopia is mixed. The main focus of this data paper is to describe the quantitative survey data since the qualitative information is not easily transmissible. However, we find it relevant to describe the mixed methodological approach. Indeed, with the diversification of life trajectories and pace of life, family structures, living arrangements, residential aspirations, or ways of working, it appears important to describe the added value of sociological and ethnographic data acquisition methods. Mixed methods are commonplace in social science. It consists in intersecting different perspectives on the same research object, i.e. to use of a diversity of methods for consolidating an explanation and strengthening the results (Aguilera and Chevalier 2021). In the current trend of research – that is interdisciplinary, pluridisciplinary – mixed-methods have gained popularity and can be used in several ways: initiation, triangulation, complementarity, development and expansion (Greene et al. 1989). Three different materials compose the *Data Domotopia*: the questionnaire data, interview transcriptions, and drawings and photographs.

The questionnaire emphasizes the dominant trends in home-making. Although precise and thorough, the collection method (web-based survey) and the mass of data implies overlooking certain subtleties such as arbitrations, housing arrangement and material adjustments, intrapersonal tensions, negotiations and conflicts of synchronicity within the household, or subtle coping strategies. The questionnaire of the *Data Domotopia* was co-designed by a transdisciplinary team of researchers (sociologists, geographer, architect, and transportation engineer). An English version of the questionnaire is available online (Pattaroni et al. 2022).

**Fig. 2:**
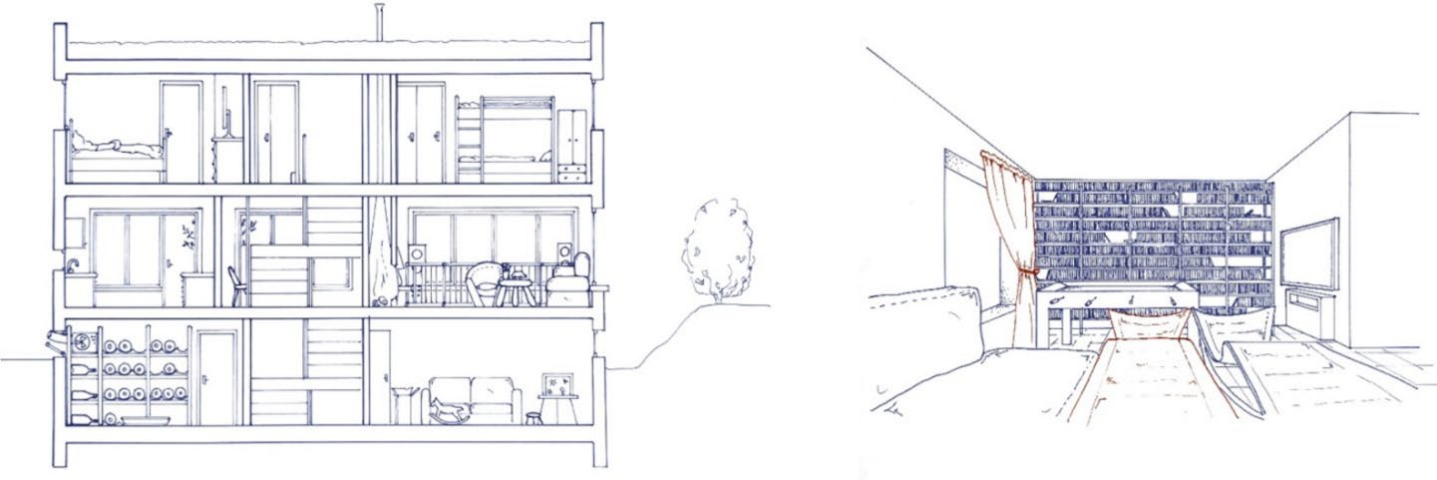
Example of cross-section on the left; and fragmented drawing of the interior on the right (interview with a 59-year-old man, 30/10/2020)

Interview transcriptions produce data without much statistical value, but yet with a high degree of detail. They are a good way to feel the situation and are leveraged in two ways. First, to fine-tune the design of the questionnaire – through a series of exploratory one-on-one interviews. The guidelines of these semi-directed preliminary interviews are available on request and are generally based on the “dwelling-dweller-context” approach described in Fig. 1. Second, for complementarity and enrichment purposes. The qualitative survey consists of face-to-face socio-ethnographic semi-directed interviews (n = 55) intentionally diverse in terms of age, professional and social positions, and residential situations. The interview guideline relies on theoretical premises ranging from action planning for spaces and objects in home-making (Breviglieri 2002; Conein 1993), to regimes of engagement (Thévenot, as described in English in Knorr Cetina et al., 2005). The interviews were preferably conducted in the dwelling of the interviewee to spatially index their comments. As shown in Fig. 2, architectural drawings are used to systematically document the spatial features of the dwellings but also the uses and appropriations observed as part of the process of domestic adaptation and transformation.

The first group of interviewees was selected among the research team’s acquaintances (n = 9). A second group was constituted from among the questionnaire respondents, focusing on individuals working from home and therefore subject to concomitant pressures related to the professional, social, or familial spheres (n = 14). Lastly, the third group of interviewees (n = 16) all share the same neighborhood, which was picked for its social and architectural diversity. The rest of the interviewees are respondents to the questionnaire who willingly shared their contact information. These were selected based on their declared time pressures. The interviews are all recorded and documented.

### Data acquisition and collection details

A market research firm was hired for administering the questionnaire. To get approximately 2500 respondents, the market research firm randomly reached out to 4500 households in the Canton of Geneva, Switzerland. Unsolicited letters were mailed, in which respondents received an invitation and an unconditional incentive of 10 SFr. Based on the experience of the market research firm, the unconditional incentive produces the highest response rate and is cheaper than phone-based surveys when conducted in Switzerland. Being able to infer an estimated response rate of a survey is of prime importance to meet the expected number of respondents with a limited time and a limited budget. If incentives have proven their efficiency in increasing the response rate (Schmidt et al., 2019), the number of pages or questions, the complexity of the posed questions, or the saliency of the survey also are determinants. Schmidt and Axhausen (2019) reviewed these determinants and propose an objective way of rating the response burden with a scoring approach.

With 108 questions, 9 transitions, and answering actions of multiple types (from simple yes/no actions to more complex tables to fill in), the response burden estimate reaches a score of 929 points for the *Domotopia* questionnaire. This score is high compared to the 65 self-administered reported surveys employed in the operationalization of the response rate estimate (ibid., p. 7).

According to the AAPOR standards (2015), the questionnaire falls under the “mail surveys of specifically named persons” in which the eligibility of all the addressees is assumed. Thus, eligible cases for which no questionnaire is filled in consist of four types of non-response: refusals and break-offs (R = 109); non-contacts (NC = 219) for which researchers receive notification that a respondent was unavailable to complete the questionnaire; others (O = 51) for which no interview is obtainable because of illness of the respondent, language barrier, or other miscellaneous reason; and unknown (U = 1859) in which nothing is known about whether the mailed questionnaire ever reached the address and thus the person to which it was mailed. Eligible cases for which the questionnaire is received fall under complete interviews (I) or partial interviews (P). Using the second AAPOR response rate formula *RR2 = (I + P) / (I + P + R + O + NC + U)*, the response rate of the questionnaire *Domotopia* is 50.4%. As no prior recruitment was done and given the high response burden, the response rate obtained is very satisfactory.

**Fig. 3:**
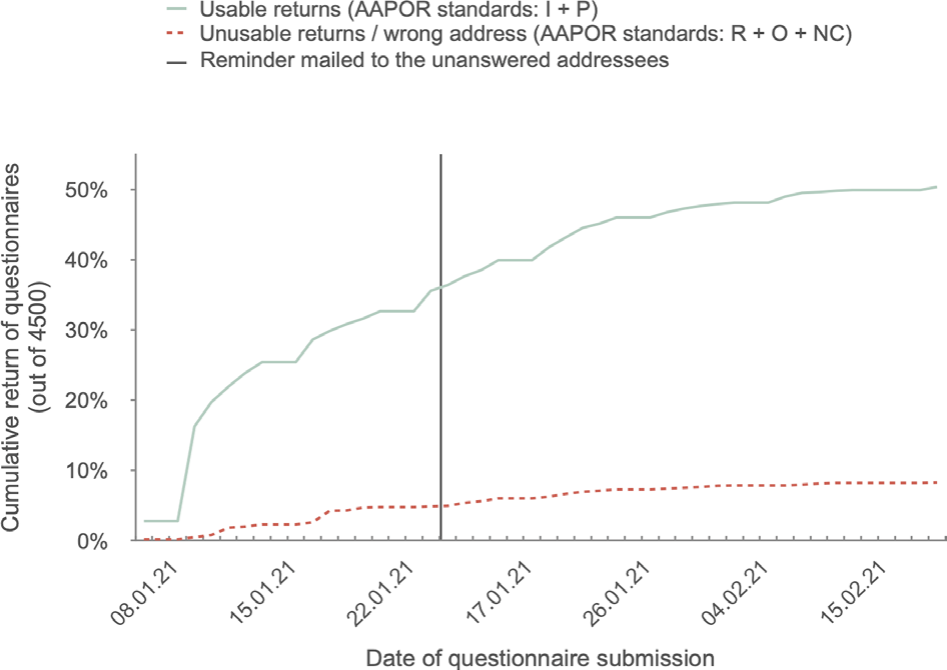
Day-by-day return of questionnaires

The return by day is displayed in Fig. 3. Approximately 25% of the questionnaires were taken in the first week, and the remaining half took 40 extra days at a slowly decreasing rate. One reminder letter was mailed three weeks after the first invitation letter. About 40% of the respondents gave consent to being contacted again for further data collection (mainly through interviews). The invitation to survey was sent with cover letters on the EPFL letterhead and included the details to access the web-based survey, and offered the opportunity to call a hotline for getting more information or a printed version of the questionnaire. Only 6.5% of the final respondents asked for a printed questionnaire, including mainly elderly respondents. The name and contact details of the person in charge of the survey were given. The addresses were aggregated at the smallest statistical sector possible, to keep as much granularity as possible, and the respondents were pseudonymized. This was done with the approval of the EPFL Ethics committee which validated the approach and the information collected.

Finally, it appears important to mention that the questionnaire was administered in the winter of 2021, when the COVID-19 pandemic temporarily transformed people’s way of living, working, and playing. In Switzerland, despite a relatively smooth management of the COVID-19 epidemiological crisis compared to neighboring countries, the winter of 2021 was quite tense. Restaurants, non-essential shops, cultural places, sports and leisure facilities were closed, and working from home was mandatory at the time. This context has to be taken into account in the interpretation of the results as the pandemic experience had consequences on home-making (e.g., see Clément et al. 2021 housing plasticity during the pandemic). Indeed, even though it was repeatedly mentioned in the questionnaire to relate to a “normal” situation and to remain within the context of “usual” daily life, some questions relating to aspirations, ideals, projections, or feelings may have been influenced by the confinement situation. The unanswered question is whether the situation is irreversible.

## Content and statistics

This section describes the data in two ways. Firstly, the topics of the questionnaire are exhaustively listed. Each topic refers to one or several questions in the questionnaire. Secondly, some descriptive statistics are provided relative to official statistics to assess the representativity of the sample.

The population in the *Data Domotopia* was sampled in the Canton of Geneva, Switzerland. It is different from the municipality of Geneva, which is only one of the 45 municipalities of the canton. Switzerland is composed of 26 cantons. The municipality of Geneva accommodates 40% of the population of the Canton of Geneva – which is one of the densest in Switzerland (approximately 500,000 + inhabitants) and one of the smallest too (OCSTAT, 2021). Geneva is a geographical area surrounded by French territory. With a significant difference in the living standards between the two countries, Geneva is an attractive territory with a high rate of daily worker immigration from France. This poses particular challenges in terms of traffic congestion. Additionally, Geneva holds an important position on the international scene with global headquarters settled in the area and a very active banking industry. As a result, 41% of the population is made of foreigners. This causes one of the biases of the *Data Domotopia* in which there is only 19% of foreigners among the respondents are Swiss. Moreover, 95% of the respondents live in Switzerland for more than 10 years. This bias is due to the address collection strategy i.e., random pick in a database maintained by the market research firm that was unlikely to capture new residents or residents with high residential mobility. Also, the questionnaire was only made available in French at the time it was administered.

### Topics of the questionnaire

The three layers of the *Data Domotopia* – as conceptualized before with the dwelling, the dweller(s), and the context – translate into some basic information, but also information about the social and residential practices. Table 2 sorts out the different topics addressed in the questionnaire with respect to the three layers.

**Table 2:** Exhaustive list of topics addressed in the questionnaire

	Basics	Social practices	Inhabiting practices
**Dwellers**	sociodemographics – backgrounds – household composition – mobility tools	professional and leisure activities – cohabitation – support network – relevant others – interpersonal tensions – domestic task allocation – time-use	time pressure – fatigue and burn out – oppression – inequalities – involvement in domestic work – lifestyle intentions
**Dwelling**	architectural basic information – house typology – housing expenses – equipment – residential status	social space – private space – sanctuary versus hyper-place* – spare rooms – house rules – equipment sharing – smartphone/computer/tech. alienation	appropriation – declared appreciation – attachment – ideals – satisfaction – sanctuarization / activation
**Context**	services and amenities – action space geography – public transit supply	social network – scheduling – mix in social spheres – free time – mobility practices	travel preferences – feel at ease – spatial familiarity – neighborhood and neighbors – sources of stress – residential aspiration

*Hyper-place: “where people, information, and communications converge” (see in Introduction). We use this word as the opposition to a sanctuary, a place preserved from the public sphere.

**Alienation to technologies refers to the growing and increasingly intense cognitive time and attention given (or taken by) digital platforms and technologies, significantly modifying lifestyles and social relationships

The “basics” provide information about the demographics, the respondent’s biography, the household composition, the basic information on architectural features and form of the dwelling, the house typology, the equipment, the monthly expenses related to housing (rent or mortgage), and lastly the morphology, accessibility, and geography in the vicinity of the dwelling.

The “social practices” refer to different spheres of life, the social relations inside and outside of the dwelling, the cohabitation rules, the scheduling of activity and trips, the space sharing and its appropriation, and more (see Table 2).

The “inhabiting practices” are rather oriented toward aspirations, attachments, perceptions, and attitudes. For example, it provides information about the symptoms of time pressure, the inequalities in task allocation between the dwellers, the feeling of ease, or the sources of stress.

Put together, these topics address several themes all closely related to the question of home-making to cope with the accelerating urban pace and time pressure pathologies. We believe that this questionnaire allows us to study the decisions and strategies related to daily mobility. In fact, it is particularly strong to delve into the spells of “immobility” – i.e. the remaining time of the day which is spent at home – and trip avoidance phenomenon.

### Descriptive statistics

The sample of the population who responded to the questionnaire tends to be representative at a 5% accuracy level in terms of gender, number of rooms per dwelling, age, and occupational status – as displayed in Table 3. Some significant biases appear in the level of education (overrepresentation of primary level i.e., compulsory education) and individual income before tax (underrepresentation of the wealthiest individuals). Other socio-demographic statistics are available in the *Data Domotopia*, such as the household financial resources, origins and nationality, occupation, etc. The spatial distribution of the population is well represented in terms of territorial typology in the outskirt of the city (outer suburbs and exurbs). Inwards, there is a noticeable imbalance between the inner suburbs (overrepresented) and the urban center (underrepresented). The distribution in household compositions is quite close to the official statistics, except for the “single” and the “other” situations. This illustrates the diversity of household compositions ranging from single- or multi-family households to non-family households. This includes for example households with several members of the same family but with no direct connection like cousins, nephews, or in-laws. Also, confusion may arise from divorced couples with children in alternating custody.

**Table 3:** Descriptive statistics and representativity of the dataset

	Domotopia 2021	Official statistics 2018–2021*
**Gender**		*(2021)*
Female	54%	52%
Male	46%	48%
+	< 1%	-
**Age**		*(2021)*
0–19	N/A	-
20–64	76%	79%
65+	24%	21%
**Occupational status**		*(2020)*
Active	60%	60.1
Retired / uncapacitated	22%	24.3
Unemployed	10%	5.0
Stay-at-home	2%	10.6
Other	5%	
Missing	1%	-
**Education**		*(2020)*
Primary	35%	27%
Secondary	26%	33%
Tertiary	39%	40%
Missing	0%	-
**Individual before tax income (CHF)**		*(2018)*
< 30’000	19%	20.4
30’000–69’999	31%	29.3
70’000–99’999	22%	17.6
100’000–149’999	14%	15.6
150’000+	8%	17.1
**Layout**		*(2019)*
1 room	2%	5%
2 rooms	5%	11%
3 rooms	16%	21%
4 rooms	30%	27%
5 rooms	28%	19%
6 + rooms	20%	15%
**Household composition**		*(2020)*
Single (1 person household)	24%	37.4
Childless couple	21%	18.7
Single-parent	7%	10.3
Couple with child(ren)	28%	29.0
Flatshare	1%	2.2
Other	19%	2.4
**Population spatial distribution**		*(2021)*
Urban center	38%	48%
Inner suburbs	27%	20%
Outer suburbs	23%	23%
Exurbs	12%	9%

* Official statistics from: Office cantonal de la statistique (OCSTAT, 2021*) and Federal Office of Statistics (*FSO, 2022*).*

The published official statistics do not have enough granularity to make a difference in this variety of cases among the 199’994 private households of the canton. In the *Data Domotopia*, the household composition presented in Table 3 is derived from question 62 “for each person living with you, please [describe their relationship to you]”, and can be double-checked by question 103 “what is your personal situation?” (see the questionnaire by Pattaroni et al. 2022).

Lastly, most of the infra-communal sectors hold at least one respondent, which provides satisfying territorial coverage. The data comes georeferenced at the official infra-communal level that covers an intermediate entity between the parcel and the municipality (SITG, 2022a). This allows full compatibility with local official statistics available in open data (OCSTAT, 2021; SITG, 2022b) while decreasing the data sensitivity in terms of privacy. By reaching out to 4500 households in the Canton of Geneva, approximately 2300 questionnaires were filled out. After data cleaning, most questions have more than 2000 exploitable answers.

## Analytical potentials and preliminary results

This section develops two themes related to the domotopia, the activity- and/or the travel behaviors. The objectives are to illustrate the wide variety of data in the survey, to call for collaborations on multiple topics, and provide intakes on the data through different use cases. The first theme provides insights into the time-space coverage of the habitual action space. Putting in perspective residential criteria alongside principles of 15-minute urbanism (Moreno et al. 2021; Kunzmann 2022), it makes visible the phenomena of residential self-selection and attitude-induced choices. The second theme focuses on the inter-personal task allocation problem. Using a major change in people’s biography – being parent – together with exhaustion symptoms in the domestic and professional spheres, it unveils preliminary results about how parenting strategies operate, and what are the effects of time pressures on the dwellers’ well-being. These two themes are drawn from four questions of the survey and discussed with additional insights from the data and the literature.

### Time-space coverage of the habitual action space

As developed in the introduction, the pursuit of stillness translates into new forms of localism in spatial development, building on the idea that proximities can alleviate a variety of saturations and urban pathologies (Antonioli et al. 2020). A few cities have started to implement or to plan the implementation of new proximities, including the city of Paris with a series of 15-minute urban interventions, and Barcelona with the developments of superblocks. In reference to the vision of Jane Jacobs (1961), these examples not only seek morphological or functional proximity, but also for social bounding, familiarity, and redeveloping a sense of neighborhood and interpersonal care. Theoretically, several urban concepts emerged. The compact cities, the neighborhood-oriented design such as the transit-oriented development, the slow-mode friendly design, or more recently the 15-minute city (Moreno et al. 2021; Kunzmann 2022), all designate a return to urban proximities. These “proximities” rapidly pose the question of what urban functions (amenities or services) should be found in the vicinity of people’s homes. And thinking further, will people be using the closest available amenity to their home, or will they be using the one that they desire even if it requires a longer trip? Therefore, there is a conceptual difference to make between the 15-minuteness of a place (morphological density and diversity) and the 15-minute lifestyle (use and aspiration of proximity).

Some of the information contained in the *Data Domotopia* allowed us to compare the time-space coverage of habitual action space with some key residential criteria rated on a Likert scale. On the one hand, a series of 6 questions (Q16A to Q16F, see Pattaroni et al. 2022) addresses the habitual action space. It describes the five places where respondents spend most of their time. As previous empirical research demonstrates that the action space displays a low degree of spatial variability, these 5 recurrent places are assumed to capture most of the locational behaviors (Isaacman et al. 2011; Schönfelder and Axhausen 2010). On the other hand, question 15 asks the respondents to score a list of 16 residential criteria in the choice of their “ideal” place of living. The criteria include functional components (e.g. accessibility and proximity to the transport network), social components (e.g. proximity to family, social diversity), and subjective components (e.g. calm, charm). These criteria capture the diversity of urban ways of living and unravel residential preferences and aspirations.

**Fig. 4:**
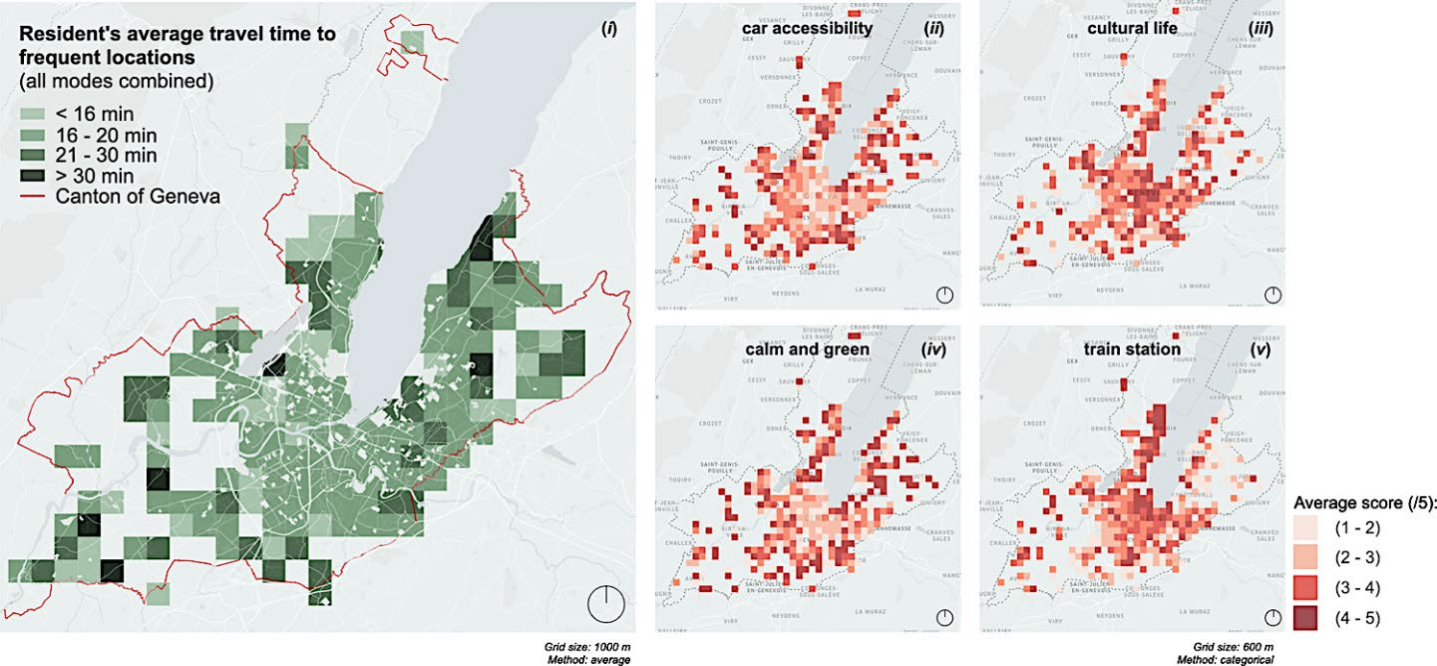
Comparison between the resident’s average travel time (i), and the average score of 4 selected residential criteria (ii), (iii), (iv), (v)

Figure 4 maps the average reported travel time to the 5 most visited locations and compares it to some of the residential criteria (importance given to car accessibility, cultural life, calm and green, and proximity to the primary and secondary train stations).

The map (*i*) displays different levels of spatial behaviors and shows that certain areas in the city are propitious to 15-minute ways of living. The expected correlation between a small action space and the closeness to the city center is not as obvious. Dense urban areas tend on average to accommodate 20- to 30-minute lifestyles, while 15-minute lifestyles are spread across the territory. This effect might be explained by the fact that 20% of the population are long-distance commuters i.e., they have a train commute to work greater than 60 min, which increases the average travel times near railway stations. Visually, some of the residential criteria tend to correlate with the spatial distribution of travel times. As displayed in Fig. 4, car accessibility (*i*) and calm and green (*iv*) are rather important criteria for residents on the outskirts. This tends to correlate positively with a higher average travel time. Conversely, the appreciation of cultural life (*iii*) is oriented inwards, which demonstrates a certain level of self-selection effect in residential location choice. The appreciation for having easy access to the train station (*v*) is characterized by a clear territorial pattern along the waterfront and down to the center of the city. This criterion is visually barely correlated with the travel time map (*i*), but also shows some level of self-selection as the pattern closely follows one of the most important railways of the country. This thread of research is deepened in another paper (see Schultheiss et al. 2022).

These descriptive preliminary results show that aspirations to different urban functions do not impact uniformly the 15-minuteness of the habitual action space. Further analyses are promising to better understand what underlies 15-minute lifestyles and stillness, be it from the perspective of residential aspirations, the perceived time pressures, or sources of adaptability.

### Task allocation problem: intra-household negotiations and change in biographies

In practice (transport planning, travel forecasting), the multi-person intra-household dependencies are barely taken into consideration in individual travel behaviors. Yet, negotiations in the domestic sphere are key in activity-travel diaries. Household negotiations for task allocation are determinant in the constraints and opportunities one might have in activity scheduling, time allocation, access – or more broadly “motility” (Kaufmann et al. 2004). As a corollary of Kaufmann and colleagues’ argument, spatial mobility is a structuring dimension of social life and permeates household negotiations. The organization of the domestic sphere is largely gendered, particularly when it comes to child care. These gender issues are rooted in the traditional schemes of masculinity and motherhood that raise questions about interpersonal relations, cohabitation, dominance and power. Previous works on involved fatherhood (Doucet 2004; Doucet and Merla 2007) analyze the possible arrangements between family and work, and show how the intra-household negotiations and the changes in professional situations can cause pathologies, such as social pressure, loss of purpose, and identity disorientation.

With the *Data Domotopia*, we can put the domestic daily task allocation in perspective with gender, with exhaustion symptoms felt in the domestic or professional sphere, and with a major change in biographies: being or becoming parents. Firstly, the respondents were asked (question 78, see Pattaroni et al. 2022) “in what areas of your life do you experience the following problems?”, including feeling overworked, fatigue, frustrations, oppression, guilt or insecurity. Note that several symptoms could be chosen for the same social sphere. Secondly, question 76 asks “who takes care of which daily task?”, including administrative tasks, cleaning, running errands, laundry making, house-related maintenance work, and cooking meals.

**Fig. 5:**
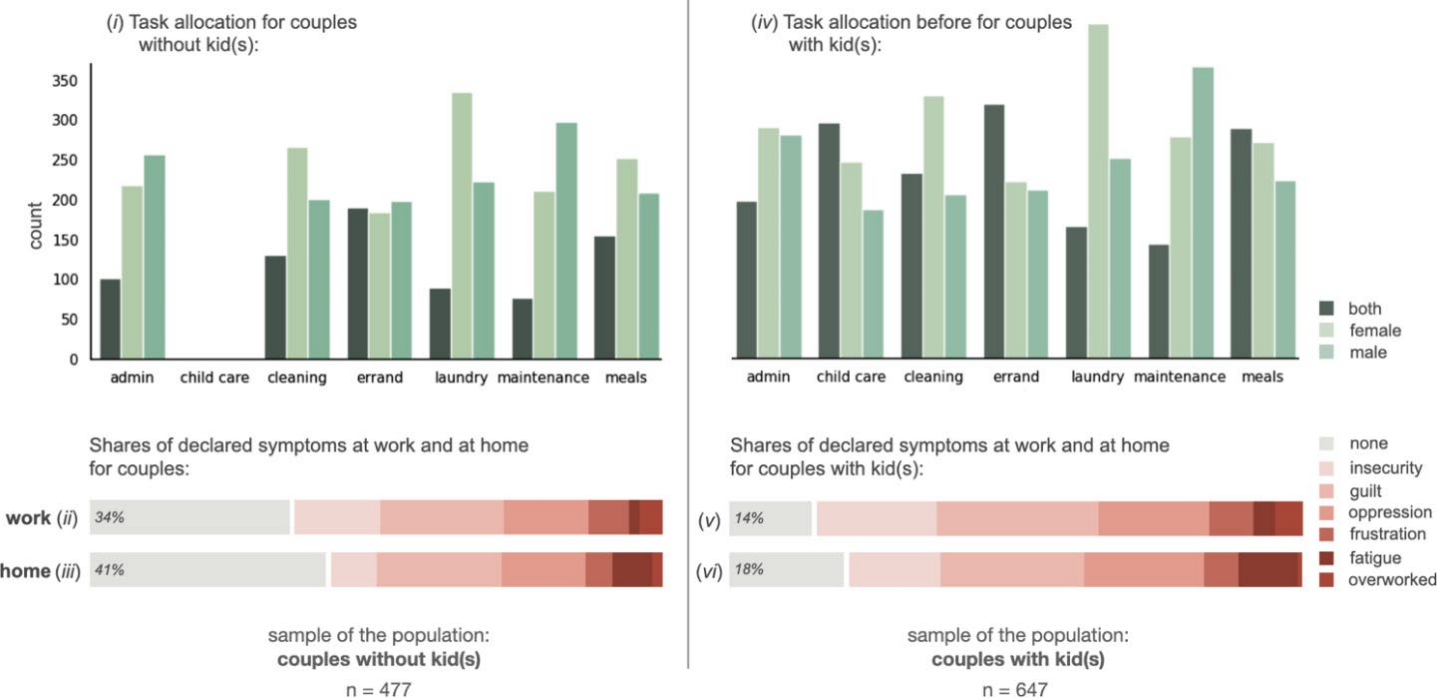
Comparison of symptoms and task allocation for couples with or without kid(s)

The respondents can indicate “who” within the household but also the support network (e.g., family, friends, external services). Figure 5 displays the comparison of symptoms and task allocation for couples without kids (n = 477), and couples with children (n = 647). Same-sex couples and non-binary couples (representing 4.2% of the population) were excluded to focus on hetero-centered normative schemes in task sharing. The stacked bar charts (*ii*), (*iii*), (*v*), and (*vi*) show the distribution of exhaustion symptoms. The bar plots (*i*) and (*iv*) display the daily tasks repartitions among the members. As shown in plots (*ii*), (*iii*), (*v*), and (*vi*), couples with no kids are less likely to declare exhaustion symptoms. At work and home, between 34 and 40% of the respondents declare having none of the mentioned symptoms. For the whole population, these shares drop to 22 and 29% respectively. Conversely, couples with kids are particularly exposed to exhaustion patterns with respectively 14 and 18% of population declaring having no symptoms. At work, the evolution of the shares of declared symptoms remains quite stable before and after kids. However, some symptoms become much more salient at home. While the feelings of fatigue, oppression and insecurity also remain quite stable before and after kids, the feelings of overwork, frustration and guilt almost double. Besides the fact that plots (*i*) and (*iv*) show a clear tendency for women to do more domestic tasks than men, the “before and after kids” comparison demonstrates that the roles of the parents in the household after the first child evolve in different manners. The division of administrative and cleaning tasks is the most stable before and after the first child is born. The cleaning also remains pretty stable, with a strong commitment from women. After the kids, the gap tends to be smaller in the equal division of cleaning (see bar “both” in Fig. 5), and there are relatively fewer households where men take full responsibility for cleaning. For the meals and errands, clear solidarity appears as more couples tend to have an equal division after the kids. This can be translated into household strategies that have a direct impact on activity scheduling and travel patterns. In terms of child care, most of couples also develop some form of equal division. Lastly, laundry and maintenance are the most gendered tasks before the kids and become even more gendered after the kids.

These first results show that exhaustion symptoms can be more or less prevalent depending on the familial configuration. Some solidarity and strategies clearly appear in the data, yet the division of housework remains highly gendered. Further analyses of the strategies within the dwelling such as the share of space and the equipment (e.g. the mobility tools, that can be obtained in question 16 C) are promising to better understand the interpersonal organization to face the pace of daily lives.

## Conclusions

The *Data Domotopia* opens up new avenues for research. It is a unique and original source of data, at the intersection of time use surveys, travel diaries surveys, surveys on living conditions, and household panels and family surveys. To the best of our knowledge, no prior examples of a similar survey exist. However, the overlaps with existing materials – as identified in Table 1 – will ease the dissemination and interpretation of the data. By reaching out to 4500 households in the Canton of Geneva, approximately 2300 questionnaires were filled out. After data cleaning, most questions have more than 2000 exploitable answers. The geocodes are aggregated at the official infra-communal level that covers an intermediate entity between the parcel and the municipality (SITG, 2022a). This allows full compatibility with local official statistics available in open data.

The *Data Domotopia* collects a wide range of topics providing both objective and subjective looks at the multidimensional aspects of home-making in a moving, liquid, fast-changing world. The overlap analysis (Table 1) reveals that the *Data Domotopia* brings not-so-common contributions regarding time pressure pathologies, the social support network (friends, family, or paid resources), and interpersonal constraints, powers, and negotiations. The tests and analysis conducted so far on the *Data Domotopia* demonstrate the richness of the data, and the quality of the information it holds – see Table 3 for the assessment of the representativity in terms of socio-demographics, territorial coverage, and household characteristics; Sect. 5 for an introductory call for collaboration about urban localism and stillness, and housework sharing; the work of Drevon et al. (2022) about domestic time pressures, and the work of Schultheiss et al. (2022) about residential aspirations are both based on the *Data Domotopia*.

By assuming that home is a central location of immobility in people’s activity-travel patterns and that home-making can reveal coping strategies and resilience practices in terms of resistance, adaptability, or transformability to make everyday life work, the *Data Domotopia* widely calls for contributions in the field of urban pathologies. For example, we identified several hypotheses that can be tested with the information held by the dataset. First, the diversification and intensification of daily rhythms lead to relevant changes in the organization of the domestic sphere. Second, the capacity to adapt to rhythmic pressures in the dwelling is determined by the level of equipment, typological changes, and social resources. And lastly, unequal exposure to rhythmic pressures, when combined with other forms of structural inequality (class, gender, race, age, etc.), leads to greater inequalities within the domestic sphere.

The themes of the *Data Domotopia* include domo-socio-spatial resources (mobility tools, habitual action space, social ease and support network); lifestyles, ideals and residential aspirations; temporal pressures, time use, organization, constraints and stress; equipment, rules and arrangements of the dwelling; domestic space and gender issues; interpersonal relations, cohabitation, dominance and power. Table 2 provides an exhaustive list of the themes and topics of the questionnaire.

Despite the epidemiological situation (COVID-19), the questionnaire was distributed in early 2021 and the first face-to-face interviews were conducted in the meantime. This context has to be kept in mind while dealing with the data, but precautions were taken in the formulation of the questions to project respondents as much as possible into a “normal” scenario.

## Data access

The data falls under the pseudonymized personal data category in the European General Data Protection Regulation (European Commission, 2022). Enough information about the respondents would allow a re-identification. Therefore, the data cannot be released as open data. However, the data will be available for research purposes, starting in 2023, in one of the SNSF-recommended data repositories.
